# Structural Dynamics of GLP-1 Analogues: Folding Energetics, Lipidation-Driven Assembly, and Aggregation Mechanisms

**DOI:** 10.3390/molecules31152556

**Published:** 2026-07-23

**Authors:** Angelo Santoro, Marco Macis, Anna Maria D’Ursi, Antonio Ricci

**Affiliations:** 1 Department of Pharmacy, University of Salerno, Via Giovanni Paolo II, 132, 84084 Fisciano, Salerno, Italy; asantoro@unisa.it; 2 Fresenius Kabi iPSUM, Via San Leonardo, 23, 45010 Villadose, Rovigo, Italy; marco.macis@fresenius-kabi.com

**Keywords:** GLP-1 analogues, peptide folding, reversible oligomerization, aggregation mechanism, multi-agonist peptides, lipidation

## Abstract

Glucagon-like peptide-1 (GLP-1) analogues are a major class of peptide therapeutics used to treat metabolic diseases. GLP-1-derived peptides are characterized by dynamic conformational ensembles in which folding, intermolecular assembly, and aggregation are strictly coupled processes. This study focused on the effects of sequence modifications, such as helix-promoting residues and backbone constraints on the helix-coil equilibrium, as well as lipidation, which creates competing equilibria among monomeric, oligomeric, and albumin-bound forms. These coupled equilibria simultaneously enhance pharmacokinetic properties and modulate conformational stability. We also explored how environmental conditions such as ionic concentration and temperature affect conformation, and emphasize how manufacturing processes act as external perturbations that could impact structural integrity. Moreover, we focus on the increasingly emerging new multi-agonist peptides, noting that their increased sequence complexity broadens conformational diversity and poses challenges to existing design methods. Despite significant experimental progress, predictive models capable of mapping the intricate interconnections among peptide sequences, lipidation patterns, and aggregation pathways remain critically limited. This highlights the importance of integrating biophysics, computation, and process science. The review points out that designing effective GLP-1 therapeutics rationally depends on managing conformational distributions across complex energy landscapes, not just stabilizing individual structures, in order to offer a new framework for developing the next generation of peptide drugs.

## 1. Introduction

Glucagon-like peptide-1 (GLP-1) is a 30-amino acid incretin hormone synthesized by proglucagon in intestinal enteroendocrine L-cells. After nutrient intake, this peptide is released into circulation, where it plays several roles, such as increasing glucose-dependent insulin secretion, inhibiting glucagon release, and inducing satiety [1,2]. These pleiotropic metabolic effects are mediated through activation of the glucagon-like peptide-1 receptor (GLP-1R), a class B1 G protein-coupled receptor widely expressed in pancreatic, gastrointestinal, and central nervous system tissues [3,4,5]. Native GLP-1 is an amidated peptide, GLP-1(7-36)NH_2_, and it is rapidly degraded by dipeptidyl peptidase-4 (DPP-4), resulting in a plasma half-life of only a few minutes [6]. The need to overcome this intrinsic instability led to the development of clinically viable GLP-1 receptor agonists, based on extensive molecular engineering aimed at optimizing proteolytic resistance, receptor potency, and systemic exposure, with partially competing structural requirements (Table 1) [7].

**Figure 1:**
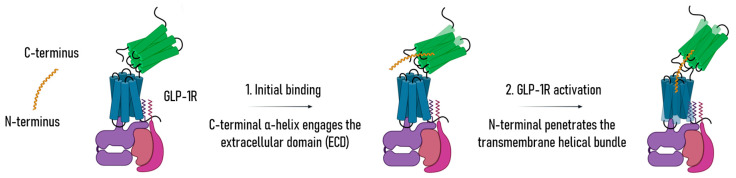
GLP-1R activation model. The GLP-1 peptide C-terminus binds the extracellular domain (ECD, green) to establish initial affinity (Step 1), while its N-terminus penetrates the transmembrane domain (TMD, blue) to trigger receptor activation and downstream G-protein coupling (Step 2). Created with BioRender.com.

GLP-1 has pronounced conformational plasticity in aqueous solution, assuming disordered and transient α-helical conformations [8,9]. In particular, nuclear magnetic resonance (NMR) studies in helix-stabilizing environments such as trifluoroethanol (TFE) mixtures, detergent micelles, or membrane-mimicking systems have shown the formation of an amphipathic α-helix, including the central ^7^Thr-Lys^28^ residues [10,11,12,13]. Interestingly, a high-resolution cryo-electron microscopy (cryo-EM) study provides a description of GLP-1 bound to its GLP-1R receptor: the C-terminal α-helix of the peptide interacts with the extracellular domain (ECD) of the receptor to establish initial affinity, then the N-terminal segment penetrates the transmembrane helical bundle to trigger receptor activation and G-protein coupling [14,15,16] (Figure 1).

**Figure 2:**
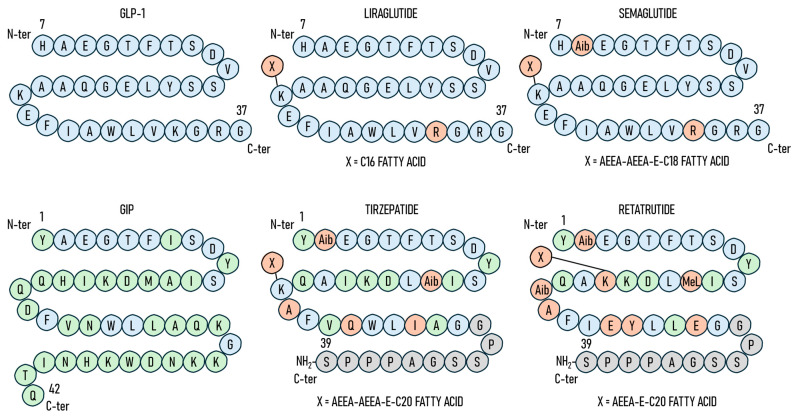
Structural comparison of native incretins (GLP-1 and GIP) and their synthetic analogues. Top row: Native GLP-1, liraglutide, and semaglutide. Bottom row: Native GIP, tirzepatide, and retratrutide. Residues are color-coded based on their structural origin and function: GLP-1-specific (blue), GIP-specific (green), non-canonical amino acids or specific substitutions (orange), and the exenatide-derived C-terminal extension (grey). X indicates the fatty acid acylation site. Abbreviations: Aib, α-aminoisobutyric acid; MeL, α-methylleucine; AEEA, aminoethoxy ethoxy acetic acid.

However, these static structures offer only a limited perspective on the binding mechanism, as they do not account for the conformational variability and dynamic selection processes occurring in solution before receptor binding [17,18]. Indeed, subtle differences in peptide conformation and dynamics within the receptor-binding pocket can lead to significant changes in signaling efficacy and biased agonism. To improve GLP-1’s poor pharmacokinetics, therapeutic analogues have been developed through sequence modifications and lipid conjugation. These modifications have been thought to alter folding propensity, protect against proteolytic degradation, and modulate systemic exposure [19,20,21,22,23]. Lipidation in particular represents a central design principle in modern GLP-1 therapeutics. Conjugation of long-chain fatty acids promotes reversible binding to serum albumin and drives concentration-dependent self-association of the peptide in solution, contributing to delayed absorption and extended pharmacokinetics [24]. Simultaneously, lipidation introduces competing equilibria among monomeric, oligomeric, and protein-bound states, thereby linking pharmacokinetics with supramolecular assembly behavior [19]. These factors influence the free-energy landscape of folding and intermolecular binding, thereby affecting both the therapeutic stability and manufacturability of lipopeptide drugs [23,25]. Recent advances in peptide engineering have further expanded the structural complexity of this class of molecules. Multi-agonist peptides targeting GLP-1R together with related receptors such as GIPR or GCGR incorporate extended sequences and additional modifications that broaden the accessible conformational ensemble (Figure 2) [26,27]. Although these molecules improve therapeutic efficacy, their structure confers greater conformational freedom, leading to increased partially folded intermediates and complicating the control of aggregation. GLP-1 analogues exhibit a complex conformational landscape, requiring both the stabilization of a single conformation and the management of conformational states through equilibria. In this review, we explore the structural dynamics of these peptides from a biophysical and pharmaceutical perspective, emphasizing mechanisms linking folding, lipidation, assembly and aggregation.

## 2. Folding Energetics of GLP-1 Analogs

**Figure 3:**
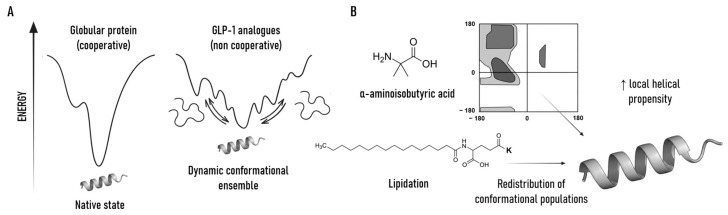
Folding energetics of incretin mimetics. (**A**) Comparative folding funnels among globular proteins and GLP-1 analogues. (**B**) Helical stabilization via sequence modifications such as Aib residues and hydrophobic anchoring.

The conformational behavior of GLP-1 and its therapeutic analogues depends on a delicate thermodynamic balance between their natural tendency to form secondary structures and environmental stability. Unlike globular proteins with cooperative two-state folding between native and unfolded states, short peptides like GLP-1 rarely exhibit distinct structures. They instead exist as dynamic ensembles with ongoing interconversion between partially folded and disordered conformations (Figure 3A) [28]. The folding behavior of these peptides results in a marginal helical stability in aqueous solution, making the conformational ensemble highly sensitive to solvent conditions, electrostatics, and intermolecular interactions [29,30,31,32].

Experimental studies consistently indicate that GLP-1 analogues populate heterogeneous conformational ensembles whose distributions are influenced by sequence modifications, lipidation, solvent composition, and the intermolecular environment [8,33,34,35] (Table 2).

Experimental studies performed by circular dichroism (CD) and NMR spectroscopy show that native GLP-1 exhibits limited α-helical content in aqueous buffers, typically on the order of 10–20% [36,37,38]. In contrast, helix-promoting environments, such as TFE or membrane-mimetic systems, can increase helicity to approximately 50–70% [12]. These studies suggest that helix formation is favored by environmental factors, which enhance intramolecular hydrogen bonding and reduce backbone-water competition [39,40,41,42]. Molecular dynamics (MD) simulations support these data by showing that GLP-1 samples a heterogeneous ensemble of partially helical conformations with rapid interconversion between alternative helical registers and bent structures [15,43,44]. Amino-acid substitutions in therapeutic GLP-1 analogues alter backbone dihedral preferences, increasing local helix propensity. In particular, the addition of the non-canonical amino acid α-aminoisobutyric acid (Aib) stabilizes α-helices by about 0.5–2.0 kcal mol^−1^ and confers resistance to DPP-4 cleavage [45,46]. Nevertheless, excessive helix preorganization can hinder binding kinetics and limit conformational flexibility needed for optimal receptor engagement, as seen in other peptide-receptor systems [17,47]. Moreover, substitutions in the sequence of GLP-1 analogues primarily serve synthetic and chemical purposes but can also influence long-range electrostatic interactions and intramolecular salt-bridge formation [48]. Among the more recent GLP-1 analogues, tirzepatide integrates sequence elements from multiple incretin hormones, including GLP-1 and glucose-dependent insulinotropic polypeptide (GIP) [49,50]. Biophysical studies show that these extended analogues have a strong tendency to form helices under physiological conditions. This suggests that stabilizing the backbone in multiple locations can offset the greater conformational flexibility that comes with longer sequences [51,52] (Figure 3B).

### 2.1. Lipidation as an Energetic Modulator

Covalent attachment of long-chain fatty acids is primarily employed to prolong the pharmacokinetic lifetime of GLP-1 analogues through reversible albumin binding [53,54]. Available experimental evidence suggests that lipidation also modifies the conformational preferences of these peptides in solution, although the magnitude of this effect is often overstated. Spectroscopic studies on liraglutide suggested that acylation preserves the amphipathic α-helical organization observed under membrane-mimicking conditions without introducing major structural rearrangements [55]. More recently, a systematic comparative analysis of five lipidated GLP-1 analogues quantified this effect using far-UV CD. In this study, non-lipidated GLP-1 displayed an estimated α-helical content of approximately 21%. In contrast, lipidated analogues exhibited values ranging from 28% to 42%, depending on both the acyl chain position and the chemical nature of the lipid moiety [23]. Importantly, the same study demonstrated that increased helicity coincides with enhanced oligomerization, making it difficult to distinguish direct intramolecular stabilization from changes in supramolecular equilibrium. Complementary molecular dynamics simulations are consistent with a model in which the lipid chain transiently interacts with the peptide’s hydrophobic regions contributing to the stabilization of compact conformational states and oligomeric assemblies. However, these observations remain model-dependent and cannot, by themselves, distinguish direct intramolecular stabilization from lipidation-driven self-association [56,57].

### 2.2. Temperature and Ionic Effects

**Figure 4:**
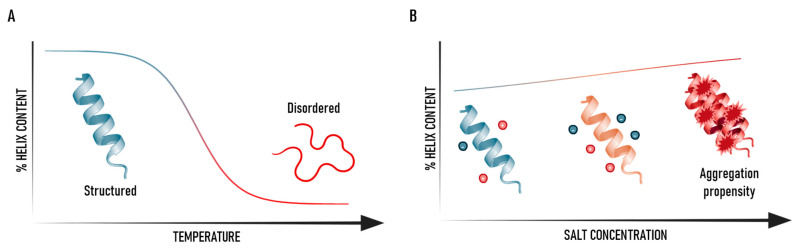
Folding energetics of incretin mimetics. (**A**) Temperature-dependent conformational changes. (**B**) Ionic strength effects on peptide–peptide interactions and secondary structure stability. Red spheres with a plus sign (+) represent positive charges/ions, while blue spheres with a minus sign (−) represent negative charges/ions.

Temperature and ionic strength influence the conformational properties of GLP-1 analogues, thereby affecting peptide stability during formulation, storage, and self-assembly. Circular dichroism studies have shown a gradual, non-cooperative decrease in helicity with increasing temperature, reflecting the marginal thermodynamic stability of GLP-1 helices in aqueous solution (Figure 4A) [58,59,60,61,62]. Conversely, moderate ionic strength partially screens electrostatic repulsion between charged residues, resulting in a modest increase in helical population (Figure 4B).

These effects are accompanied by changes in intermolecular interactions, making secondary structure and self-association intrinsically coupled phenomena rather than independent processes. In GLP-1 therapeutics, this structural plasticity directly influences the balance between functional oligomerization and irreversible aggregation. Experimental evidence demonstrates that pH modulates oligomer size distribution, secondary structure, and aggregate morphology, while elevated temperature accelerates assembly rearrangement and aggregation. In particular, even subtle changes in lipidation site or fatty-acid chemistry profoundly reshape the self-assembly landscape, leading to distinct oligomeric architectures and stability profiles [23,25,57,63]. Likewise, combined computational and experimental studies have shown that environmental perturbations that alter the conformational ensemble of GLP-1 analogues can destabilize native helical states while increasing the population of aggregation-prone conformations [8,64]. These observations emphasize that controlling temperature and solution composition is primarily relevant for formulation development and long-term physical stability, where small environmental perturbations can significantly alter self-assembly and aggregation behaviour [65,66,67,68,69,70].

### 2.3. Coupled Folding and Receptor Binding

One of the most physiologically significant examples of GLP-1 folding energetics is the receptor binding, which reflects a key case of coupled folding and binding equilibria. Structural studies of GLP-1 receptor complexes obtained by cryo-electron microscopy demonstrate that peptide agonists adopt well-defined α-helical conformations when bound to the receptor (Figure 1) [15,17,18]. Current structural and computational evidence is consistent with contributions from both conformational selection and induced-fit mechanisms [47,71,72,73]. Recent cryo-electron microscopy studies complemented by three-dimensional variability analysis and molecular dynamics simulations have considerably expanded this view by demonstrating that receptor activation remains intrinsically dynamic after peptide binding. In this study, although semaglutide and taspoglutide establish interaction networks highly similar to those of native GLP-1, they stabilize distinct dynamic states of the receptor, particularly within the extracellular domain and extracellular loops. These differences are not apparent in static cryo-EM structures but emerge from three-dimensional variability analysis and MD simulations, suggesting that ligand efficacy is influenced not only by the final bound conformation but also by the receptor-ligand complex’s dynamic behaviour [74]. Recent structural studies on retatrutide have further reinforced this concept. By combining high-resolution cryo-EM with molecular dynamics simulations, Li et al. showed that triple agonism is associated with ligand-specific conformational rearrangements extending beyond the orthosteric binding pocket. In particular, subtle differences in peptide-receptor interactions propagate toward the extracellular loops and transmembrane helices, generating receptor conformational ensembles that differ among GLP-1R, GIPR and GCGR. These findings support the emerging concept that peptide efficacy depends not only on receptor affinity but also on the ability of individual agonists to modulate receptor dynamics selectively [75]. Therefore, successful engineering of next-generation GLP-1 analogues requires balancing conformational preorganization with enough structural plasticity to preserve proper receptor dynamics.

## 3. Lipidation and Conformational Equilibria

Lipidation is a key structural modification typical of synthetic GLP-1 agonists. Lipidation affects the biophysical behavior of GLP-1 agonist peptides by favoring reversible intermolecular assembly and stabilizing albumin binding (Table 3). As discussed in Section 2.1, current experimental evidence indicates that lipidation induces only a moderate increase in α-helical population [23]. Therefore, the effect of lipidation is not the stabilization of a single folded state, but rather a redistribution of the conformational ensemble toward states that favour reversible self-association, protein binding and, potentially, interactions with biological membranes.

### 3.1. Structural Design Principles of Lipidated GLP-1 Analogues

The first clinically used GLP-1 analogue, liraglutide, was engineered with a C16 palmitoyl chain linked via a γ-glutamyl spacer to lysine in position 26. This modification preserves the amphipathic helical core of GLP-1, introducing a hydrophobic tail capable of reversible self-association and albumin binding. The initial rationale focused on delayed renal clearance and decreased proteolysis. However, later structural studies showed that acylation also influences peptide–peptide interactions and helps stabilize conformation [76]. For this reason, the lipidation in semaglutide was further optimized by introducing a C18 fatty acid linked via a hydrophilic spacer [77]. Structural analyses indicated that a longer acyl chain increases albumin affinity and mitigates excessive aggregation, favoring reversible assembly dynamics [22].

### 3.2. Coupled Intra- and Intermolecular Equilibria Induced by Lipidation

**Figure 5:**
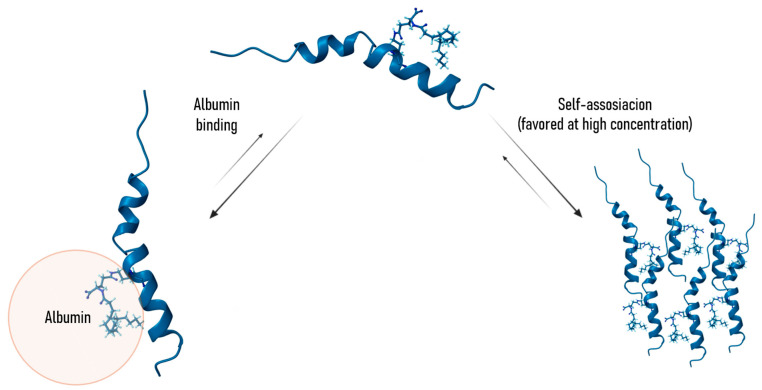
Schematic representation of the concentration-dependent equilibrium of GLP-1 analogues. At low peptide concentrations (**left**), binding to albumin is favored via a reversible interaction. At high concentrations (**right**), the monomer undergoes self-association into reversible oligomeric forms.

Several investigations have shown that the conformational effects of lipidation are relatively modest. NMR studies of liraglutide in solution demonstrated that acylation preserves the amphipathic α-helical segment observed in native GLP-1 in helix-promoting solvent conditions. Chemical shift perturbations experiments demonstrated that lipidation induces local backbone modification in the region near the lipidation site [55]. Similarly, circular dichroism analyses of several therapeutic GLP-1 analogues showed only moderate increases in α-helical content upon lipidation, suggesting that fatty-acid conjugation shifts the equilibrium among pre-existing conformational states [23,56]. Computational studies provide a molecular interpretation of these observations. According to MD simulations, the fatty-acid chain remains highly dynamic and undergoes transient contacts with hydrophobic residues along the peptide backbone. Rather than forming persistent intramolecular interactions, these contacts generate short-lived compact conformations that reduce solvent exposure of the lipid moiety while preserving substantial backbone flexibility [56]. The same hydrophobic interactions that transiently stabilize compact monomeric conformations also promote intermolecular association. Small-angle X-ray scattering (SAXS), size-exclusion chromatography coupled with multi-angle light scattering (SEC-MALS), analytical ultracentrifugation, and spectroscopic studies demonstrated that lipidated GLP-1 analogues reversibly assemble into concentration-dependent oligomeric species, with oligomerization driven predominantly by cooperative interactions among fatty-acid chains together with electrostatic contacts between peptide backbones [23,63]; for example, in liraglutide it has been observed that lipidation is responsible of the formation of reversible heptameric structures in solution [57,78]. Albumin binding introduces a second competing intermolecular equilibrium. Structural and biochemical studies have established that the fatty-acid chain inserts into hydrophobic binding pockets of serum albumin while leaving the receptor-binding surface of the peptide largely solvent-accessible, thereby preserving biological activity [79,80,81,82,83,84]. Because the lipid chain cannot simultaneously participate in peptide self-association and albumin binding, these two processes compete for the same hydrophobic motif. Experimental investigations performed under conditions mimicking the subcutaneous compartment demonstrated that albumin binding predominates at low peptide concentrations. In contrast, at higher local concentrations, such as in subcutaneous depots, self-association becomes increasingly favorable (Figure 5). This indicates that conformational equilibria are dynamically distributed among monomeric, oligomeric and protein-bound states [63].

Moreover, advanced analysis of liraglutide in pharmaceutical formulations using asymmetric flow field-flow fractionation (AF4) combined with multi-detection has validated the existence of specific oligomeric distributions while maintaining native aggregation states [85].

### 3.3. Lipidation in Multi-Agonist Peptides

**Figure 6:**
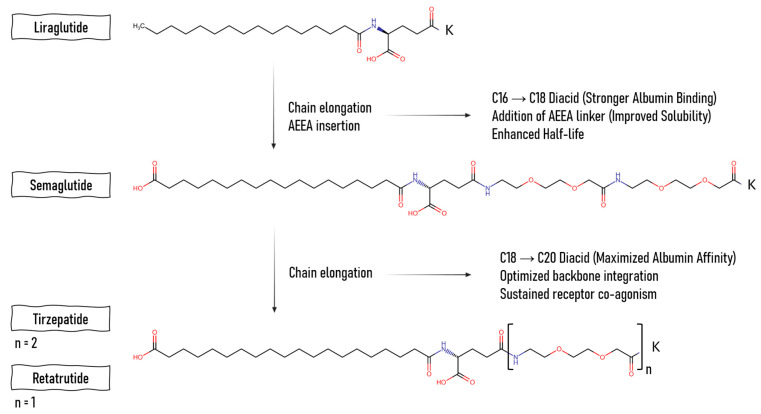
Lipophilic modifications of GLP-1 and multi-agonist analogues. Comparison of the fatty acid chains and linker for liraglutide, semaglutide, tirzepatide, and retratrutide.

The design principles established for lipidated GLP-1 analogues have recently been extended to multi-receptor agonists, in which lipidation is integrated with extensive sequence engineering to optimize receptor activity, pharmacokinetics and metabolic efficacy simultaneously. Next-generation therapeutics such as tirzepatide and retatrutide exemplify this integrated design approach. In tirzepatide, the structural implications of lipidation become more complex; indeed, this molecule incorporates a C20 fatty diacid moiety attached through a linker optimized for albumin binding while maintaining dual GIP/GLP-1 receptor activity. A substantial α-helical content and a preserved receptor-binding geometry are indicated by biophysical analyses, suggesting that lipidation does not compromise the folding of receptor-interacting domains [51,86]. In retatrutide, the integration of three receptor agonist motifs necessitates careful positioning of the lipid chain to avoid steric interference with receptor engagement. Although detailed high-resolution structural data remain limited, biochemical characterizations confirm that lipidation preserves receptor potency and confers extended half-life, suggesting maintenance of conformational integrity [26,87]. Thanks to these features, lipidation functions as both a pharmacokinetic modifier and a structural stabilizer, helping reduce excessive flexibility in elongated backbones (Figure 6). Nevertheless, the transition from single-receptor GLP-1 analogues to multi-agonists has introduced additional design constraints, as peptide engineering must simultaneously balance receptor selectivity, conformational stability, pharmacokinetic optimization and intermolecular interactions [86,88]. Consequently, future progress will depend less on empirical optimization and more on quantitative models capable of separating the contributions of lipidation, sequence engineering and receptor-specific interactions.

## 4. Aggregation Mechanisms in GLP-1 Lipopeptides

In contrast to folding energetics and lipidation-driven self-assembly, the molecular mechanisms of aggregation in GLP-1 analogues have only recently been elucidated, thanks to solution biophysics and computational modelling. Therefore, current evidence derives from the characterization of reversible oligomeric species rather than from direct observation of irreversible aggregation pathways. This distinction is important because the supramolecular assemblies detected under physiological or formulation conditions differ fundamentally from the heterogeneous aggregates associated with physical instability (Table 4). As reported in Section 3, over the last decade, complementary analytical techniques have shown that lipidated GLP-1 analogues form well-defined oligomeric reversible assemblies whose abundance depends on concentration, formulation conditions, and lipid architecture. These assemblies remain reversible and preserve the α-helical secondary structure indicating that oligomerization represents a functional supramolecular equilibrium rather than a manifestation of pathological aggregation [23,57,78,89,90].

Frederiksen and colleagues provided the initial detailed structural analysis of liraglutide oligomerization. They demonstrated that the peptide mainly forms heptameric structures, stabilized by cooperative interactions between fatty-acid chains and electrostatic contacts among neighbouring peptide molecules. These assemblies exhibit dynamic structural fluctuations but preserve a hydrophobic core formed by the palmitoyl chains [57]. Recent native ion mobility mass spectrometry (IM-MS) and large-scale MD simulations demonstrated that GLP-1 analogue oligomerization is not a simple sequential process but a dynamic network of competing pathways that lead to closely related low-energy states. This assembly is driven primarily by the highly flexible lipidated residue, which explores the peptide surface to establish hydrophobic contacts, and is stabilized by intermolecular salt bridges between charged amino acids. Moreover, extensive conformational heterogeneity was shown at both monomeric and oligomeric levels [91]. The coexistence of compact and more expanded conformers suggests that GLP-1 oligomers should be regarded as dynamic ensembles that interconvert on experimentally accessible timescales. This interpretation is highly consistent with previous SAXS observations and with MD simulations, which similarly describe lipidated GLP-1 analogues as sampling broad conformational landscapes [23]. Further evidence supporting this dynamic model has recently been obtained through electron-capture dissociation coupled with direct mass spectrometry and MD simulations, which resolved previously hidden oligomerization pathways inaccessible to conventional ensemble techniques [92]. Current evidence supports a unified model in which lipidated GLP-1 analogues exist as dynamic supramolecular systems characterized by reversible exchange between monomeric, oligomeric and albumin-bound species. This framework is strongly supported by multiple orthogonal experimental techniques and provides the most robust structural description currently available for therapeutic GLP-1 analogues. In contrast, the molecular events responsible for initiating irreversible aggregation remain largely unresolved. Available experimental studies primarily describe either native oligomeric populations or the final aggregated material, whereas transient intermediates linking these two states have not yet been directly observed. For this reason, the relationship between reversible self-assembly and pathological aggregation remains one of the major unanswered questions in the biophysics of GLP-1 therapeutics.

## 5. Manufacturing and Analytical Control of Conformational Integrity

Conformational integrity of therapeutic GLP-1 lipopeptides depends not only on their intrinsic sequence but also on manufacturing, purification, solid-state handling, and formulation conditions. Since these peptides exist on shallow energy landscapes, even slight process-related disturbances can cause shifts toward partially unfolded or aggregation-prone states (Table 5). Therefore, process development and analytical control should be viewed as strategies to stabilize structure. Throughout manufacturing, process parameters such as temperature, solvent composition, ionic strength, and shear stress are key structural variables. Since α-helical segments in GLP-1 analogues have limited thermodynamic stability, even minor deviations can impact conformational balance. Real-time solution monitoring through process analytical technology (PAT) can help detect early signs of aggregation or conformational changes. Consequently, manufacturing must be seen as an extension of structural design: sequence engineering influences the protein’s tendency to fold; lipidation promotes supramolecular assembly; and process control maintains these carefully engineered equilibria during production and storage.

### 5.1. Synthetic Strategies and Their Structural Consequences

Most GLP-1 analogues are produced via solid-phase peptide synthesis (SPPS) followed by solution-phase lipidation or convergent coupling. Each synthetic step exposes the growing peptide chain to conditions such as acidic cleavage, base-mediated deprotection, and oxidative environments. These can cause chemical modifications that impact folding behavior. Chemo-enzymatic methods have been investigated to improve sustainability and minimize by-products in the production of GLP-1 analogues, for example, in synthesizing liraglutide [93]. From a manufacturing standpoint, reducing side reactions such as racemization, aspartimide formation, or oxidation is critical, as these defects directly lead to a significant decrease in overall yield, substantial challenges in purifying closely related impurities, and a consequent increase in production costs. Beyond these primary process-economic concerns, suppressing such side reactions is also vital from a structural perspective; it prevents the formation of structurally disturbed impurities that may promote peptide aggregation or compromise conformational stability. Even small changes in sequence can affect helix stability or disturb amphipathic balance, highlighting the need for high chemical fidelity. Convergent synthetic methods, supported by temporary metal complexation, have shown enhanced coupling efficiency for lipidated intermediates [94]. These methods might reduce conformational disorder at intermediate stages, thereby enhancing downstream purification and solid-state stability.

### 5.2. Purification and Resolution of Conformational Variants

High-performance liquid chromatography (HPLC) is essential for purifying GLP-1 analogues. Nonetheless, reversed-phase techniques primarily separate molecules based on hydrophobicity rather than on conformational differences. To improve the separation of similar peptide species, electrostatic repulsion-reversed phase (ERRP) has been introduced, which alters the balance between charge and hydrophobicity [95]. Chromatographic separation can distinguish between variants such as oxidized, deamidated, or misacylated forms that differ slightly in their conformational properties from the target molecule. Isolating these variants is crucial because even small impurities can change aggregation kinetics. Importantly, purification procedures must avoid causing conformational changes. Factors such as high levels of organic solvent, extreme pH, or high temperatures during chromatography can temporarily destabilize helical regions. Therefore, process optimization aims to find conditions that provide high resolution without compromising the native-like secondary structure.

### 5.3. Control of Native Aggregation During Formulation

As we discussed before, lipidated GLP-1 analogues tend to form reversible oligomers. During formulation, the objective is to preserve this controlled assembly without permitting progression toward irreversible aggregation. Gold standard technologies like dynamic light scattering (DLS), size-exclusion chromatography (SEC), CD, and native mass spectrometry allow orthogonal evaluation of aggregation state and secondary structure. However, these techniques may suffer sensitivity limitations or require the use of stationary phases that may destroy or stimulate the presence of aggregates. For these reasons, more recent technologies have gained interest in recent years, such as AF4 coupled with multi-detection [85] or microfluidic modulation spectroscopy (MMS), which allow recording the native state of a peptidic system with high sensitivity without perturbing equilibrium [96]. Accordingly, all these techniques allow monitoring peptidic systems during stress testing, thermal cycling, agitation, and freeze-thaw exposure, providing kinetic data on their aggregation onset. Such studies define operational stability windows and inform excipient selection. Buffers are chosen not only for pH control but also for ionic strength and specific ion effects that stabilize α-helical conformations [97].

## 6. Conclusions and Future Perspectives

GLP-1 analogues are a class of therapeutic peptides whose biological activity, pharmacokinetics, and stability are determined by a carefully balanced conformational landscape. Instead of settling into a single dominant structure, these molecules exist as dynamic ensembles influenced by equilibria involving folding, lipidation-driven assembly, and environmental interactions. A key conclusion of this review is that these processes are interconnected and should be understood as features on a common free-energy surface (Table 6).

While folding energetics and lipidation-driven self-assembly are now relatively well characterized, aggregation remains the least understood component of this landscape. The current understanding is limited by the lack of direct experimental observations of aggregation intermediates and by reliance on ensemble-averaged measurements. Consequently, the mechanisms of aggregation in GLP-1 lipopeptides are best viewed as kinetically accessible escape routes from metastable conformational ensembles rather than precise, deterministic processes. Several key challenges arise from this view. First, existing structural models depend heavily on static receptor-bound conformations and averaged spectroscopic data, offering limited insight into the transient states that influence folding, assembly, and aggregation. Second, the connection between lipid-driven oligomerization and aggregation remains unclear, especially under conditions relevant to manufacturing, storage, and administration. Third, there is a lack of predictive frameworks that connect sequence, lipidation chemistry, and aggregation propensity, with current design approaches remaining largely empirical. These issues are compounded in next-generation multi-agonist peptides, where increased sequence length and modular architecture introduce additional structural variables that are expected to challenge the experimental and computational characterization of conformational ensembles (Table 7).

This trend is reflected by the growing pipeline of multi-receptor peptide agonists, including compounds such as VK2735 and CT-868, which follow related engineering principles but for which detailed structural and biophysical characterization remains comparatively limited [98,99,100]. Understanding how sequence modularity and lipidation together influence these conformational ensembles is vital for advancing peptide drug design. Progress will depend on combining experimental and computational methods capable of capturing diverse conformations across various scales. Multiscale molecular simulations, when validated against experimental data, can effectively identify rare yet important states. Additionally, techniques such as native mass spectrometry, hydrogen-deuterium exchange, and high-resolution separation are crucial for detecting low-abundance intermediates and analyzing oligomeric diversity. It is also important to consider how manufacturing processes, such as synthesis, purification, and formulation, alter environmental conditions, thereby shifting conformational equilibria and affecting aggregation risks. Factors such as ionic strength, interface exposure, and solid-state organization should be considered core elements of the structural landscape rather than external variables. Ultimately, designing GLP-1-based therapies will require a shift in approach: instead of stabilizing single conformations, strategies should aim to modulate the overall distribution of conformational states within the ensemble. This perspective offers a unified framework for connecting sequence design, lipidation, and process conditions to functional outcomes and stability. While achieving predictive control of these complex systems is challenging, it presents a valuable opportunity to advance peptide therapeutics.

## Figures and Tables

**Table 1:** Comparison of structural and pharmacological properties between GLP-1 and modified analogues.

Feature	Native GLP-1	Therapeutic Analogues
Length	~30 amino acids	30–40+ amino acids
Conformational state	Disordered ensemble	Partially preorganized ensemble
Helical content (aqueous)	Low (~10–20%)	Moderate (sequence-dependent)
Stability	Rapid degradation (DPP-4)	Protease-resistant
Pharmacokinetics	Minutes	Hours-days
Key modifications	None	Substitutions, lipidation
Binding mechanism	Induced folding upon receptor binding	Coupled folding-binding with preorganization

**Table 2:** Physicochemical factors that govern helix-coil equilibria in GLP-1 peptides. Upward arrows (↑) indicate an increase or enhancement, while downward arrows (↓) indicate a decrease or reduction.

Factor	Mechanism	Effect on Helicity	Thermodynamic Contribution
Amino acid substitutions	Backbone restriction	↑ Helicity	↓ Entropy (unfolded state)
Solvent (TFE, micelles)	Reduced water competition	↑ Helicity	↑ Enthalpic stabilization
Temperature	Increased backbone motion	↓ Helicity	↑ Entropy
Ionic strength	Charge screening	Slight ↑ Helicity	↓ Electrostatic repulsion
Lipidation	Intramolecular hydrophobic contacts	Apparent ↑ helicity	Redistribution of ensemble

**Table 3:** Multiscale effects of lipidation on GLP-1 analogues. Lipidation simultaneously modulates intramolecular conformational sampling, intermolecular assembly, and protein binding equilibria.

Level	Effect	Mechanism	Outcome
Intramolecular	Local compaction	Hydrophobic contacts	Reduced flexibility
Intermolecular	Oligomerization	Acyl chain interactions	Reversible assembly
Membrane interface	Membrane association	Fatty acid insertion	Increased local peptide concentration
Protein binding	Albumin association	Fatty-acid insertion	PK extension
Global energetics	Ensemble redistribution	Shift in equilibrium	Modulation of structural populations

**Table 4:** Distinction between functional oligomerization and pathological aggregation.

Property	Functional Oligomers	Aggregates
Structure	α-helical	β-sheet-rich
Reversibility	Reversible	Irreversible
Driving forces	Hydrophobic + electrostatic	Backbone H-bonding
Kinetics	Fast equilibrium	Nucleation-dependent
Role	PK modulation	Instability
Conditions	Physiological	Stress conditions

**Table 5:** Impact of manufacturing and processing steps on the conformational landscape of GLP-1 lipopeptides.

Process Step	Perturbation	Structural Impact	Risk
SPPS	Chemical stress	Sequence defects	Aggregation seeds
Purification (HPLC)	Solvent/pH extremes	Helix destabilization	Conformational drift
Lyophilization	Dehydration	Solid-state rearrangement	Polyamorphism
Formulation	Ionic environment	Equilibrium shift	Aggregation
Handling	Shear/interfaces	Partial unfolding	Nucleation

**Table 6:** Integrated representation of the coupled conformational equilibria governing GLP-1 lipopeptides.

State	Structural Features	Driving Forces	Coupled Equilibria	Functional Role
Disordered monomer	Low helicity, high flexibility	Conformational entropy	Folding ⇄ partially folded states	Reservoir for binding and assembly
Partially folded monomer	Transient α-helices	Intramolecular H-bonding	Folding ⇄ binding ⇄ oligomerization	Binding-competent conformations
Receptor-bound state	Stable α-helix (ECD + TM engagement)	Binding enthalpy + folding coupling	Folding-binding coupling	Signal activation
Lipid-stabilized monomer	Locally compacted structure	Hydrophobic intramolecular interactions	Folding ⇄ oligomerization ⇄ albumin binding	Increased stability, PK tuning
Oligomeric assembly	α-helical, ordered multimers	Hydrophobic + electrostatic interactions	Monomer ⇄ oligomer ⇄ albumin-bound	Controlled depot formation
Albumin-bound state	Constrained conformation	Fatty-acid insertion into albumin	Monomer ⇄ albumin-bound ⇄ oligomer	PK extension, protection
Aggregation-prone intermediate	Partially unfolded, β-component	Backbone exposure	Loss from all equilibria	None (off-pathway)
Aggregates (β-sheet)	Cross-β structure	Intermolecular H-bonding	Irreversible endpoint	Loss of function

**Table 7:** Structural and biophysical features of clinically relevant GLP-1 analogues.

Peptide	Key Modifications	Structural Effect	Self-Assembly Behavior	Albumin Binding	Aggregation Propensity
GLP-1 (native)	None	Low intrinsic helicity	Minimal	None	Low (but condition-dependent)
Liraglutide	C16 lipid (Lys26), Lys34→Arg	Moderate helix stabilization	Reversible oligomers (heptamers)	Strong	Low under physiological conditions
Semaglutide	C18 diacid (AEEA)_2_ (Lys 26), Ala8→Aib, Lys34→Arg	Increased helicity, rigidity	Reversible oligomers (more stable)	Very strong	Very low (high stability)
Tirzepatide	C20 diacid (AEEA)_2_ (Lys20), Ala2→Aib, Ala13→Aib, hybrid sequence (GLP-1/GIP)	Distributed helix stabilization	Likely oligomerization (less defined)	Strong	Moderate (sequence complexity)
Retatrutide	C20 diacid (AEEA) (Lys17), Ala2→Aib, Ala13→MeL, Ala20→Aib, Multi-agonist (GLP-1/GIP/GCGR)	Extended helical segments	Poorly characterized	Strong	Moderate (sequence complexity)

## Data Availability

No new data were created or analyzed in this study. Data sharing is not applicable to this article.

## Abbreviations

The following abbreviations are used in this manuscript:

GLP-1	Glucagon-like peptide-1
GLP-1R	Glucagon-like peptide-1 receptor
DPP-4	Dipeptidyl peptidase-4
NMR	Nuclear magnetic resonance
TFE	Trifluoroethanol
ECD	Extracellular domain
TMD	Transmembrane domain
Aib	α-aminoisobutyric acid
MeL	α-methylleucine
AEEA	Aminoethoxy ethoxy acetic acid
CD	Circular dichroism
MD	Molecular dynamics
GIP	Glucose-dependent insulinotropic polypeptide
SAXS	Small-angle X-ray scattering
SEC-MALS	Size-exclusion chromatography coupled with multi-angle light scattering
AF4	Asymmetric flow field-flow fractionation
IM-MS	Ion mobility mass spectrometry
PAT	Process analytical technology
SPPS	Solid-phase peptide synthesis
HPLC	High-performance liquid chromatography
ERRP	Electrostatic repulsion-reversed phase
DLS	Dynamic light scattering
SEC	Size-exclusion chromatography
MMS	Microfluidic modulation spectroscopy
